# *Planta* Centennial editorial

**DOI:** 10.1007/s00425-026-05165-x

**Published:** 2026-10-08

**Authors:** Dorothea Bartels, Matthew H. K. Cheng, Stefan de Folter

**Affiliations:** 1 IMBIO, University of Bonn, Kirschallee 1, 53115 Bonn, Germany; 2 Springer Nature, Europaplatz 3, 69115 Heidelberg, Germany; 3 Advanced Genomics Unit (UGA-Langebio), Centre for Research and Advanced Studies (Cinvestav), Km. 9.6 Libramiento Norte, Carretera Irapuato-León, 36824 Irapuato, Guanajuato Mexico

## *Planta*: a century of plant biology research and beyond

In 2025, *Planta* reached its 100th year of publication. This occasion prompted the journal to look back on its journey, reflect on key research it has published, and to look ahead to how plant biology research is and will continue to evolve.

While *Planta* is now an international journal, it began as a German-language periodical named *Planta:* *Archiv für Wissenschaftliche Botanik* (translated “Planta: Archive for Scientific Botany”). Led then by Wilhelm Ruhland (Leipzig, Germany) and Hans Winkler (Hamburg, Germany), the journal published its first issue on 24 February 1925. The issue featured 4 articles which reported on *Beggiatoa mirabilis* cell permeability​ (Ruhland and Hoffmann 1925), influence of chemical agents in starch content and osmotic parameters of stomata cells ​(Arends 1925)​, distribution of geotropic sensitivity in negative geotropic plant organs ​(Herzog 1925)​, and plasma membranes ​(Weis 1925)​. In 2025, *Planta* published 304 articles (Clarivate 2026).

The journal’s growth was largely helped by a decision after the second World War to explicitly welcome manuscripts written in additional ‘congress’ languages, or English (https://archive.org/details/sim_planta_1948_36/page/n1/mode/1up). A shift started with the first English article in 1957 ​(Cooil and Bonner 1957)​ and completed with the official renaming of the journal to *Planta: An International Journal of Plant Biology* in 1971 (https://link.springer.com/journal/425/volumes-and-issues/100-1). This transition enabled *Planta* to publish and disseminate research on a global level.

To mark its Centennial, *Planta* invited a series of Perspectives to reflect on key research published in the journal. While *Planta* is interested in all aspects of plant biology, an important focus has been on photosynthesis and metabolism, hormones and signaling, discoveries in botany, and molecular mechanisms in plant biology. These perspectives have been collected in the “Centennial Collection” published in 2025 and 2026.

## Current state of *Planta*

The focus of *Planta* has always been to report experimental approaches which lead to new discoveries in plant science. The subjects encompass the main areas of plant science and range from metabolism, plant hormones as internal and external signals, development, to new discoveries of plants with special adaptation mechanisms to their habitat. The last hundred years have seen major changes in methodologies and experimental approaches. Early publications used mainly microscopy and physiology, then biochemical experiments were often included, and now genetics and molecular biology often combined with bioinformatics are tools which lead to the discovery of mechanisms and large data sets. The history of *Planta* is marked by two different categories of publications, those which address experimentally general plant metabolism and its regulation by intrinsic and environmental factors, and those which explore the diversity of the plant kingdom and investigate unique adaptation mechanisms to ecological niches.

## The Centennial Collection

The broad range of the subjects and the wide scope of experimental approaches is mirrored in the selection of the perspective manuscripts compiled in the Centennial Collection: photosynthesis, metabolism, hormone signaling in developmental and environmental processes, unique plant phenotypes, and application of genetics and molecular biology in plant science. All publications discussed in the perspective articles have been highly cited, and the authors are excellent scientists who made major contributions to plant science research and beyond. The perspectives are written for a general readership, and hopefully attract readers who enjoy reflecting on the history and consequences of discoveries. But hopefully these articles also catch the attention of young scientists who are not familiar with the research topics.

Discoveries in plant metabolism have been an important focus of *Planta* and the following perspectives feature some milestones.

Two perspectives discuss photosynthesis and stomatal function (von Caemmerer and Farquhar 2025; von Caemmerer et al. 2025). The authors contributed substantially to understanding the complex metabolic fluxes in photosynthesis. They established a mathematical model to capture the carbon acquisition of plants and to understand biochemical reactions of leaf photosynthesis. Their original publications in *Planta* (Farquhar et al.^1980^; von Caemmerer and Farquhar 1981) belong to the most highly cited articles published in *Planta*. These studies led to a detailed understanding of the regulation of carbon dioxide diffusion in leaves. The work on gas exchange in plant leaves and the connection to the biochemistry of photosynthesis led to optimization in plant breeding, and their gas diffusion model is applied in modern gas exchange measurement systems world-wide. The models still form a foundation for present photosynthesis research which is featured in the perspectives by Walker and Schroeder (2026) and by Stitt (2026). Walker and Schroeder discuss the role of photorespiration in the context of energy balancing under changing environmental conditions. Models have been developed to follow energy fluxes in different metabolic settings. The knowledge gained in these investigations could lead to improve plant productivity under challenges of climate change. However, Walker and Schroeder also point out that more genome scale metabolic studies are necessary to fully understand photorespiratory fluxes and the influence of plant productivity. Stitt (2026) highlights transgenic plant approaches and how these investigations contributed to the understanding of the role of Rubisco and other enzymes of photosynthesis in plant energy metabolism. Stitt emphasizes that plant transformation allowed to generate antisense plants with a progressive decrease of the central enzyme Rubisco. This made it possible by the group of Mark Stitt and others to study the role of Rubisco in the energy metabolism which determines plant productivity. Understanding the energy network triggered by Rubisco and other Calvin cycle enzymes is important in optimizing photosynthetic performance of agricultural plants grown under field conditions.

*Planta* has been an important journal to report new discoveries which were revealed by studying unexpected or unusual plant phenotypes. It turned out that several of these discoveries have a potential for practical applications. Four important examples are reviewed in the perspectives by Bartels et al. (2025), Barthlott (2026), Kiss (2025) and Routaboul et al. (2025). Bartels et al. (2026) refer to the first molecular analysis of a desiccation tolerant resurrection plant *Craterostima plantagineum* (Bartels et al. 1990). This publication triggered interest in studying desiccation tolerance in plants on the molecular and biochemical level by various research groups. All the studies, including recent detailed genomic and transcriptomic analyses, show that desiccation tolerance is a complex network of many changes on the genomic, transcriptomic and metabolomic level. It also illustrates what may be possible to translate into plant productivity during periods of drought.

One of the most highly cited *Planta* articles is the discovery of the so-called “Lotus Effect” (superhydrophobic self-cleaning plant surfaces) (Barthlott and Neinhuis 1997), The report on the “Lotus Effect” attracted a lot of attention in botany but even more in materials sciences and physics. The perspective by Barthlott (2026) describes the changes triggered by the discovery of the phenomenon of superhydrophobicity in understanding plant surfaces which exhibit extreme water repellency. This perspective highlights the cellular structures, physics and technical applications of the “Lotus Effect”.

Colors of flowers, fruits, seeds, tubers or leaves determine processes or properties in plants. Often flavonoids are responsible for the colors of plant organs. Flavonoids belong to the large group of polyphenols and are synthesized in diverse enzymatic networks. Changes in flavonoid patterns and the dissection of the biosynthetic flavonoid pathways have contributed to important biological discoveries and to breeding plants with modified properties. Important steps in flavonoid biology are highlighted in the perspective by Routaboul et al. (2025). The authors look back to the discoveries of flavonoid metabolism and emphasize the potential of plant-based flavonoid metabolites in biotechnological applications from food production and plant breeding to medical applications.

Plants sense gravity, which makes shoots grow upwards and roots grow downwards. Sensing gravity is a key process in the life cycle of plants. There have been several, often controversial hypotheses about the nature of gravity sensing mechanisms. Early models for gravitropism have been deduced from research on the unicellular *Chara* rhizoid and the multicellular cress roots (Sievers and Schröter 1971; Sievers and Volkmann 1971). John Kiss, one of the main researchers in the field of gravitropism, reviews the different approaches which have been taken to identify the nature of gravity sensing cells in his perspective article (Kiss 2025). A main step in dissecting gravity sensing was the analysis of mutants and space flight experiments which present novel insights into gravitropism until today.

Hormonal pathways are crucial for plant development. Over the years, many studies related to elucidating their functions have been published in *Planta*. One central plant hormone is auxin. Roni Aloni is one of the pioneering researchers in the auxin field; he has been using immunolocalization of auxin molecules and the now well-known *DR5* reporter line already in his early studies. The perspective by Aloni (2025) describes how auxin induces and controls vascular differentiation and venation pattern formation in leaves. Furthermore, Aloni provides an overview of the involvement of auxin in floral organ pattern formation.

The molecular response to auxin is mediated by a group of transcription factors called Auxin Response Factors (ARFs). The most-studied member of this family is MONOPTEROS (ARF5/MP), and Berleth et al. (2026) discuss the importance of MP in axis formation during embryogenesis and plant development, including vein pattern formation described by Aloni (2025). Berleth and colleagues describe how in the early 1990s, they, and other developmental geneticists, stimulated by the Drosophila field, used big genetic screens to identify mutants leading to the cloning of key genes for plant development. Interestingly, they also describe the gap that existed at the time between geneticists and developmental biologists, and the skepticism they received on their work that MP could be mediating the canalization of auxin flux (Przemeck et al. 1996).

The hormone abscisic acid (ABA) is well-known for its role in seed development such as seed maturation and inhibition of germination. Nguyen et al. (2026) look back on the original work by the group of Maarten Koornneef, one of the pioneers of Arabidopsis genetics, who genetically dissected the maternal and zygotic origins of ABA during seed development (Karssen et al. 1983). By using genetics, they discovered that dormancy induction by ABA primarily depends on zygotic tissue. Recently, deeper insights have been obtained on ABA transport and biosynthesis using genetics and single-cell transcriptomics Nguyen et al. (2026).

In contrast to ABA, the hormone gibberellin (GA) stimulates seed germination. GA signaling induces activation of cell-wall weakening enzymes in the endosperm. Peter Hedden, an authority in the field, tells us about the establishment of two protocols that were important for later studies of the role of GAs in seed germination (Hedden 2025). One bioassay was based on induction of α-amylase activity in the aleurone of seeds, and the other was based on the use of a tensiometer to measure the force required to break through the endosperm. The first work was developed by Russell Jones with Joseph Varner in the US at the end of the 1960 s, and the second work by Steven Groot in the group of Cees Karssen at the end of the 1980s, who was a plant physiologist and also worked at the Wageningen University as did Maarten Koornneef. Despite the many discoveries, there are still numerous open questions about GA signaling in seed germination (Hedden 2025).

Also Reactive Oxygen Species (ROS) have now been recognized as important signaling molecules. Originally ROS were thought to be toxic by-products, now it is known that these molecules are involved in many signaling processes during plant development. Graham Noctor provides a historical overview of the H_2_O_2_-metabolising systems (Noctor 2025). It was in the mid-1970s that a pathway was proposed that linked the molecules ascorbate and glutathione to the reduction of H_2_O_2_ (Foyer and Halliwell 1976). The ascorbate–glutathione pathway was first characterized as a chloroplastic antioxidative process, now also known as the Foyer–Halliwell–Asada pathway. Finally, it is illustrated that plants have multiple pathways for ROS removal (Noctor 2025).

The retrospective articles in the Centennial collection illustrate the history of major developments in plant science, but what will determine plant science in the future? New experimental methods and techniques have been developed and their application in studying plants will advance and perhaps change our understanding of plant science. Three examples have been chosen and are illustrated in perspective articles.

Plants respond to environmental cues and when adequate lead to flowering. George Coupland, an authority in the field of flowering control, takes the reader on a journey from discoveries made in the 1920s until today and his vision on future directions for the field (Coupland 2026). Discoveries were made from how plants measure day length, the nature of the florigen, and how plants respond to a winter period. The first discoveries were made in the field of plant physiology, followed by genetics and molecular biology. Current and future studies will use protein biochemistry, genomics, imaging, and mathematical modeling to refine our understanding of the mechanisms of floral induction. The exploitation of this knowledge will have a strong influence on plant breeding in the future.

The availability of plant genome and transcriptome sequences revealed that alternative splicing often occurs in plants and presents a possibility for adaptation to changing environmental conditions. The importance of alternative splicing and the metabolic consequences are described by Toh et al. (2025). The authors describe the methodologies used for studying alternative splicing and they illustrate how alternative splicing is caused by different stressors. Alternative splicing seems to be more frequently found in plants in response to stress than in animals. Toh et al. (2025) point out that alternative splicing may be exploited in the future to select plants which are more resilient to stress caused by climate change or biotic challenges.

The development and establishment of refined methodologies for lipid research is illustrated by Gutbrod and Dörmann (2026). Lipids as essential components of plant cells present important energy sources, but they also act as central signaling molecules, and form lipid bilayers as structural cellular components. The investigations of the regulation of lipid synthesis and lipid functions require sophisticated analytical tools for the analysis of the different lipid classes. Gutbrod and Dörmann (2026) describe the state-of-the-art of lipidomics technologies and how the applications open new avenues in understanding plant metabolism.

Looking forward, we hope that the next 100 years of *Planta* will be exciting times. Continuation of discoveries in the plant science field with the use of new molecular and analytic techniques, the use of sophisticated and sensitive equipment or the introduction of new model species will lead to new and sometimes unforeseen discoveries. We will observe how the integration of artificial intelligence with research will influence scientific publications and progress in science. We are convinced that scientific dissemination will continue to be important and we welcome your contributions to *Planta*.

We thank all authors who contributed to the Centennial collection for their excellent and very valuable contributions. It was an enjoyable experience to look back and to learn which routes science took until today and where it may go.
